# 40 Years of Research Put p53 in Translation

**DOI:** 10.3390/cancers10050152

**Published:** 2018-05-21

**Authors:** Virginie Marcel, Flora Nguyen Van Long, Jean-Jacques Diaz

**Affiliations:** Univ Lyon, Université Claude Bernard Lyon 1, INSERM 1052, CNRS 5286, Centre Léon Bérard, Centre de Recherche en Cancérologie de Lyon, 69008 Lyon, France; floranvl@gmail.com (F.N.V.L.); jeanjacques.diaz@lyon.unicancer.fr (J.-J.D.)

**Keywords:** p53, translational control, ribosome, cancer

## Abstract

Since its discovery in 1979, p53 has shown multiple facets. Initially the tumor suppressor p53 protein was considered as a stress sensor able to maintain the genome integrity by regulating transcription of genes involved in cell cycle arrest, apoptosis and DNA repair. However, it rapidly came into light that p53 regulates gene expression to control a wider range of biological processes allowing rapid cell adaptation to environmental context. Among them, those related to cancer have been extensively documented. In addition to its role as transcription factor, scattered studies reported that p53 regulates miRNA processing, modulates protein activity by direct interaction or exhibits RNA-binding activity, thus suggesting a role of p53 in regulating several layers of gene expression not restricted to transcription. After 40 years of research, it appears more and more clearly that p53 is strongly implicated in translational regulation as well as in the control of the production and activity of the translational machinery. Translation control of specific mRNAs could provide yet unsuspected capabilities to this well-known guardian of the genome.

## 1. Introduction

The *TP53* gene codes the canonical p53 protein, which had been discovered as a cellular protein of 53 kDa co-immunoprecipitating with the large T antigen of SV40 virus in 1979 [1,2]. Using biochemical and molecular approaches, p53 protein was defined as a transcription factor in the early 1990s [3]. This protein is composed of a N-terminal transactivation domain (TAD) involved in the recruitment of transcriptional co-factors, a central DNA-binding domain (DBD) directly interacting with DNA in a sequence-dependent manner through specific p53-response elements, and a C-terminal domain of oligomerisation (OD), the p53 transcription factor being active as tetramer [4]. Historically, p53 was first described as an oncogene until it was discovered that the cloned version of p53 corresponds to a mutant form [3]. Since then, its function was tied up to its activity in stress response (radiation, carcinogen, ribonucleotide privation, oncogenic activation…). From its discovery, it was indeed known that p53 is stabilized and activated in response to stress in order to regulate transcription of numerous genes promoting cell cycle arrest, apoptosis, senescence or DNA repair, p53 being rapidly known as the “guardian of the genome” [5]. Addition of post-translational modifications inhibits interaction of p53 with the E3-ubiquitin ligase Hdm2 that promotes proteasome-dependent degradation in basal condition thus ensuring the maintenance of low p53 protein level in most of human cells. Based on the role of p53 in maintaining genome integrity in response to genotoxic and oncogenic stresses, p53 has been defined as a tumor suppressor [6]. This was supported by additional observations. First, more than 50% of human tumors carry a *TP53* mutation [7,8]. Second, all p53-null mice develop spontaneous tumors within their first year of life [9]. Finally, germline *TP53* mutation is associated with a familial syndrome, the Li-Fraumeni Syndrome, which is characterized by development of multiple tumors at early onset [10,11]. Intriguingly, recent studies performed in mice suggest that p53 tumor suppressor activity is not strictly dependent upon major p53 effector pathways, such as cell cycle arrest and apoptosis, as expected [12,13,14]. However, it is still not clear whether its tumor suppressor activity could result from the additional biological cellular functions that have been recently linked to p53, including for example metabolism, maintenance of stem characteristics, reproduction or aging, to cite only some of them [15]. Moreover, it has to be said that depending on the nature of the *TP53* mutation, mutant p53 can exhibit opposite biological effects thus increasing the difficulty to draw a clear view of p53 activities [16]. Altogether, the view of p53-related biological functions has largely evolved since the discovery of p53 and there is no doubt it will continue.

One of the remaining questions in the field is how a single protein is able to integrate the myriad of internal and external stimuli to finely regulate cell-fate by selecting the appropriate genes within the repertoire of its target genes? Several mechanisms have been proposed. Most, if not all, rely on the high diversity of p53-responsive elements present in promoters of p53-target genes that differentially interact with distinct p53-containing complexes [16]. Indeed, p53 protein is subjected to numerous post-translational modifications (phosphorylation, acetylation, ubiquitination, sumoylation, neddylation, methylation…) that not only promote p53 stabilization but also confer p53 specificity to some target genes. Moreover, specificity of p53 binding to some target genes can also result from interaction with several proteins, including p53 isoforms or viral proteins [17,18]. However, in addition to directly regulate transcription, it appears that p53 alters the translational regulation of some mRNAs, thus adding a novel layer by which p53 can finely modulate gene expression [19,20,21,22].

While only few studies have been dedicated to understanding the role of p53-mediated translational regulation in the fine modulation of gene expression driven by p53, the relationship between p53 and translation has been largely reviewed to illustrate different facets of p53 biology since: (i) an increase in p53 mRNA translation contributes to p53 activation in response to stress [23]; (ii) alteration of ribosome biogenesis corresponds to one of the stresses able to activate a p53-dependent response (i.e., nucleolar or ribosomal stress) [24,25]; (iii) usage of alternative translation start sites allow expression of different p53 isoforms modulating p53 transcriptional activity [23,26,27]; and (iv) antibiotics targeting the translational machinery could restore wild-type p53 transcriptional activity from nonsense p53 mutant [28,29,30]. In a previous review, we discussed how translation contributes to tumor suppressive of p53 [31]. In the present review, we will focus on the molecular mechanisms by which p53 regulates translation of specific mRNAs. Following a brief overview of the basics of translational regulation and its involvement in cancer, we will discuss the role of p53 in translational reprogramming, either through direct or indirect intervention, to attempt discerning whether p53-dependent translation of some specific mRNAs could contribute to the p53-mediated regulation of gene expression.

## 2. Translation and Cancer

### 2.1. Regulation of Global Protein Synthesis and Cancer

**Figure 1:**
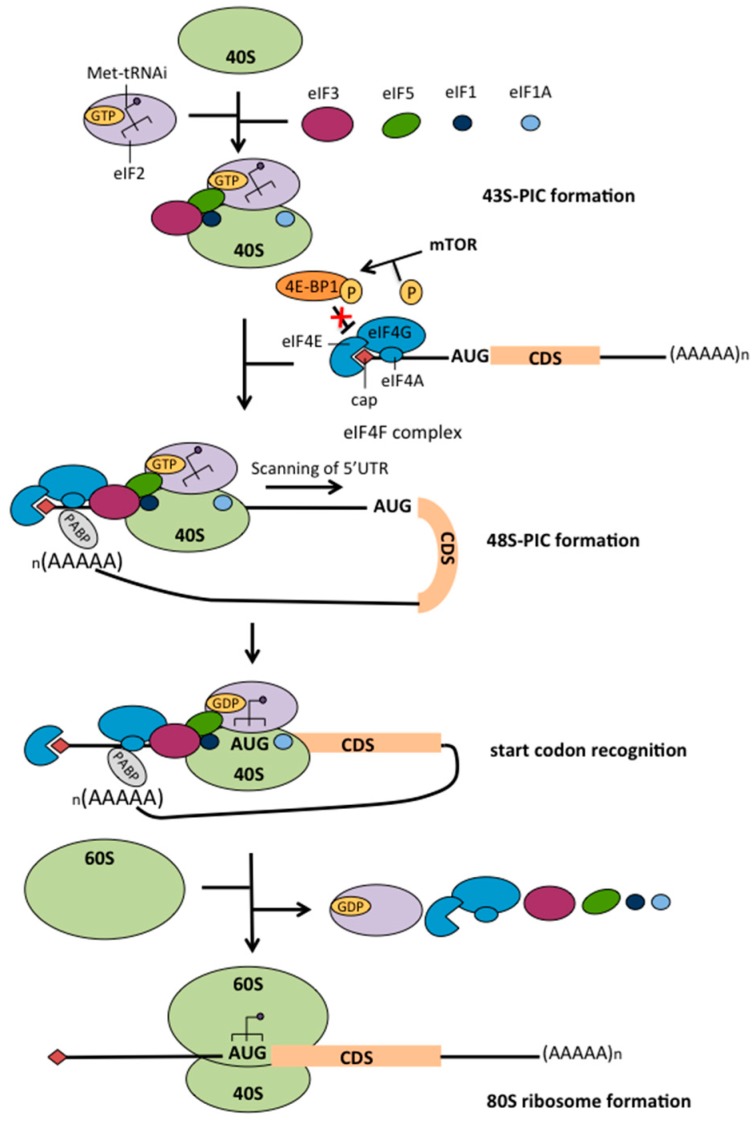
Overview of the cap-dependent translation initiation. Cap-dependent translation initiation can be divided in four main steps: (1) 43S-PIC formation, (2) 48S-PIC assembly, (3) start codon recognition and (4) formation of the 80S mature ribosome. The Met-tRNAi-bound to eIF2 and several eIFs (1, 1A, 3 and 5) are recruited to the 40S small sub-unit ribosome to form the 43S-PIC (1). The 43S-PIC is recruited to the cap of the mRNA through the interaction with the eIF4F complex in order to form the 48S-PIC (2). The eIF4F complex is composed of three proteins: the cap-binding protein eIF4E, the RNA helicase eiF4A and the scaffolding protein eiF4G that allows 43S-PIC binding to the mRNA cap through eIF3. Formation of eIF4F complex is mainly regulated by mTOR signaling pathway, mTOR phosphorylating the translation repressor 4E-BP1 that inhibits eIF4F complex formation through the binding of eIF4E. Moreover, a «closed loop» structure of the mRNA is formed through the interaction of the PABP to the mRNA poly (A) tail to increase translation initiation efficiency by favoring the binding of eIF4E to the cap. Then, the 48S-PIC scans through the mRNA 5′UTR until the start codon (3). The start codon is recognized by the Met-tRNAi that induce the hydrolysis of eIF2-GTP bound and release of eiF2-GDP and the other eIFs (1, 3, 5 and eIF4F). The large subunit 60S of the ribosome will then join the 40S ribosome to form the 80S ribosome and start translation (4). (AAAAA)_n_: poly (A) tail; CDS: mRNA coding sequence; eIF: eukaryotic initiation factor; Met-tRNAi: initiator-methionyl non-coding transfer RNA; PABP: poly (A) binding protein; PIC: pre-initiation complex; 4E-BP: 4E-binding protein. Figure adapted from [32].

Translation is one of the last steps of gene expression as it allows protein synthesis from mRNA. One of the major control of protein synthesis occurs during the initiation step of translation, which corresponds to formation of the mature human 80S ribosomes and binding of the initiator-methionyl non-coding transfer RNA (Met-tRNAi) to the mRNA start codons (Figure 1). Since translation of the vast majority of cellular mRNAs requires presence of a chemical modification at their 5′-end (i.e., m^7^GpppN, also called the cap structure) that is bound by eukaryotic initiation factors (eIFs) to recruit ribosome, cap-dependent initiation is generally considered as the main mechanism regulating translation of all mRNAs at once (for reviews see [32,33]). Cap-dependent translation is finely regulated in response to stress (irradiation, heat-shock, nutriment privation…) by several signaling pathways, including PI3KT/AKT/mTOR and RAS/MAPK for example, that modulate eIFs activity [34]. For example, mTOR (mammalian target of rapamycin) activates the eIFs binding to cap structure thus promoting cap-dependent translation through the phosphorylation of several downstream effectors including 4E-BP1 (4E-binding proteins) [35,36]. Indeed, the non-phosphorylated 4E-BP1 form binds to eIF4E, directly competing with eIF4G for eIF4E binding, and thus inhibits the formation of eIF4F complex. In addition to these classical signaling pathways regulating the eIFs, quantitative variation of ribosomes and tRNAs can directly impact global protein synthesis. Indeed, ribosome biogenesis and global protein synthesis are closely and dynamically regulated to accommodate cell growth and proliferation [37,38]. For instance, MYC, a protein exhibiting pro-proliferative and pro-survival activities, promotes protein synthesis of cancer cells by favoring synthesis of all the components of the intrinsic translational machinery, ribosomal RNA (rRNA), ribosomal proteins and tRNAs [39].

Expression and activity of many eIFs are increased in various cancers suggesting that cap-dependent translation initiation is increased in cancer [40,41]. Interestingly, several studies showed that targeting of the cap-recognition complex with direct inhibitors of eIFs or inhibitors of upstream signaling pathways decreases tumor growth and overall exhibit anti-cancer activity [42]. In addition to eIFs, the role of global protein synthesis resulting from increase in ribosome biogenesis has been well documented in cancer. Nucleoli are the sites of ribosome production located within the nucleus and, as early as 1896, increase of nucleoli size and number has been observed in cancer cells [40]. It has been rapidly proposed that hypertrophic nucleoli reflect an increase in ribosome biogenesis and protein synthesis rate that would be necessary to sustain cancer cell growth and proliferation [43]. High protein synthesis rate in cancer cells as a cause or consequence of cell transformation was equivocal for a long time, until a pioneering study reported the direct role of increased ribosome biogenesis coupled with a high protein synthesis rate in tumorigenesis [44]. In this elegant study, the elevated global protein synthesis rate driven by *Myc* oncogene observed in *Eμ-Myc* lymphomagenesis mice was reduced by introducing a mutation in the ribosomal protein L24 (*RPL24*) gene. Reduction of protein synthesis rate blocked cell growth, cell proliferation and tumorigenic potential, demonstrating the important role of protein synthesis hyperactivation in tumorigenesis. This observation was supported by recent studies showing that inhibiting ribosome biogenesis using RNA polymerase I inhibitors (responsible of rRNA transcription) or platinum-based drugs such as oxaliplatin, whose—unexpectedly—main mechanism involves defect in ribosome production, specifically kill cancer cells [45,46,47]. Overall, these data suggest that cancer cells are «addicted» to elevated protein synthesis rate. These discoveries not only offer novel therapeutic strategies for cancer treatment but also demonstrate the role of increased global translation in cancer. The role of p53 in regulating global protein synthesis has been previously reviewed, including one in the present issue [48]. Several studies indeed reported the role of p53 in inhibiting global protein synthesis, either by inhibiting ribosome biogenesis or cap-dependent translation through inhibition of eIF4E transcription, formation of eIF4E: eIF4G complex via its target gene TRIM22, or phosphorylation of 4E-BP1 [31,49,50,51,52]. 

### 2.2. Regulation of Specific mRNA Translation and Cancer 

It is now admitted that in addition to alteration of global protein synthesis, alteration of specific mRNA translation corresponds to one of the strategies allowing cancer cells to rapidly adapt and survive in unfavourable conditions. Regulation of specific mRNA translation involves regulatory elements located within the mRNA itself, termed *cis*-regulatory elements [53]. In the 5′UTR of mRNAs, upstream open reading frames (uORFs) can be found that correspond to small open reading frames located upstream the main ORF. The exact function of uORFs is not yet totally deciphered however it appears very often that uORFs are translated by ribosomes whose release avoid initiation at the downstream start codon of the main ORF thus repressing its translation [54]. Interestingly, uORFs are often located in genes coding growth factors and oncogenes and overcoming uORF inhibitory effects gives advantage to cancer cells to favour their growth and proliferation by molecular mechanisms involving an interplay between several regulatory elements of the mRNA and of the translational machinery [55]. For example, binding of HuR (Hu antigen R) and hnRNPA1 (heterogeneous nuclear ribonucleoprotein A1) proteins to the 3′UTR mRNA of the proto-oncogene *HER2* in cancer cells overcome the inhibition of the repressive uORF located in the 5′UTR of HER2 mRNA, thus contributing in HER2 over-expression in some breast tumors [56]. In addition to uORFs, sequence-specific RNA elements are found in the 3′UTR of the mRNAs that are bound by microRNAs (miRNAs). miRNAs are 20 bp-long non-coding RNAs (ncRNAs) that bind to their complementary target within mRNAs in a sequence-specific manner to promote their translational repression followed by their degradation [57]. Several mechanisms have been proposed by which miRNAs reduce translation of their target mRNAs, including decreased ribosome recruitment to mRNA, blockage of translation elongation or increase in ribosome release [58]. In cancer, miRNA can act either as oncogenic miRNAs (oncomiRs) that are up-regulated to repress tumor suppressor expression, or as tumor suppressor miRNAs that are downregulated to favour expression of oncogenes [59].

Other trans-regulatory factors have been identified including RNA-binding proteins (RBPs) that bind sequence or structural motifs located in the UTR of targeted mRNAs. Up to date, about 400 RBPs have been described that present diverse functions including regulation of translation initiation [60]. Among the structural motifs bound by RBPs, the Internal Ribosome Entry Sites (IRES) is the most studied. Around 10% of cellular mRNAs are predicted to contain IRES that require protein binding for regulating translation, including the mRNA encoding p53 [61,62]. These particular RBPs are termed ITAFs (IRES Trans-Acting Factors) and allow the recruitment of eIFs or ribosomes to the mRNAs [63,64]. Not only expression of ITAFs is altered in cancers but a switch from cap-dependent translation to IRES-dependent translation has been observed, altogether favouring expression of oncogenes promoting tumorigenesis and progression [40].

Exciting studies have recently showed that ribosomes and tRNAs are not “housekeeping complexes” that are passively involved in translation, but are direct regulators of specific mRNA translation favouring tumorigenesis. Indeed, ribosomes can present different compositions that are associated with distinct translational activity, this concept being known as the “specialized ribosome” concept [65]. The source of ribosome diversity results from the 80 ribosomal proteins (RPs) (stoichiometry, post-translational modifications, paralog incorporation…) or the 4 rRNAs (post-transcriptional modifications such as 2′-O-ribose methylations, pseudo-uridylations or base modifications). For example, RPS25-containing ribosome has been shown to coordinate translation of mRNA subset encoding proteins involved in vitamin B12 signaling [66]. Moreover, it has been reported that depending on the abundance of particular tRNA, translation of specific mRNAs enriched with the corresponding cognate codon can be selectively increased to participate in metastasis formation for example [53,67,68]. Altogether, it appears that like transcriptional or epigenetic regulation of gene expression, selective translation plays a key role in tumor initiation and progression.

## 3. Evidences from “Omic” Data for a Role of p53 in Regulating Translation

### 3.1. Genome-Wide Approaches Dedicated to Analysis of Translation 

The emergence of genome-wide approaches dedicated to the analysis of translational regulation has allowed increasing interest of translational control in different physio-pathological contexts, including cancer [53]. Two main approaches have been developed [69]. First, polysome profiling coupled either to microarray or RNA-seq compares the distribution of each individual mRNA between cytoplasm and polysomal fractions (i.e., ribosome-bound mRNAs that are actively translated), in two biological conditions [70,71]. Second, the most recent approach termed ribosome profiling (or Ribo-seq) compares the protection by ribosomes of each individual cytoplasmic mRNAs quantified by RNA-seq [72]. These two approaches allow quantification of change in translational efficiently for each individual mRNA. Moreover, they are always coupled to classical analysis of cytoplasmic mRNA steady-state levels that is not only used as genome of reference to take into account expression of particular isoforms for example, but also to compare transcriptional and translational rate. However, in contrast to polysome profiling, Ribo-seq also gives qualitative information regarding precise location of ribosomes along mRNAs thus allowing the identification of novel translational initiation sites, regulatory elements in non-coding sequences or open-reading frames induced by frameshift events. Altogether, these approaches had opened up novel roads in the understanding of translational regulation in particular biological conditions, including those in response to p53 activation. 

### 3.2. p53 Activation Affects Translation Efficiency of Some Specific mRNAs

Up to now, only four studies investigated the role of p53 in translational regulation using “omic” approaches (Table 1). Most of these studies used the same strategy. Endogenous wild-type p53 is activated using Nutlin-3a, a small molecule inhibiting p53/Hdm2 interaction by specific binding to Hdm2 that inhibits p53-dependent proteasome degradation and activates p53 transcriptional activities [73]. However, each study used different cell lines as well as different concentrations and time-points (Table 1). Despite these different experimental conditions, it appears that p53 activation was always associated with a change in translational efficiency of numerous mRNAs that were not associated with changes in transcription and/or stability [19,20,21,22]. In addition, Zaccara and colleagues showed that Nutlin-3a treatment did not affect gene expression at transcriptional or translational levels in MCF7-shp53 cells, thus clearly demonstrating that change in translation in response to Nutlin-3a treatment is dependent upon p53 [21]. 

When compared to cytoplasmic mRNA steady-state levels, the significant alteration in translational efficiency accounted for about 25 to 50% of the p53-associated change in gene expression, with an equivalent increase and decrease in translation efficiency (Table 1). Overall, these studies indicate that, as expected, p53 activation is associated with a massive transcriptional reprogramming, accounting for 75–50% of p53-induced change in gene expression. However, in response to p53 activation, they showed an unexpected translational reprogramming of 25–50% of genes whose transcription and mRNA steady-state level are not altered by p53 activation. 

The highest change in translation efficiency has been observed by comparing wild-type p53 and p53-null HCT-116 cells in response to serum starvation [19]. Translation is a high-consuming energy task, which is finely tuned to adapt the pool of translational machinery to the cell needs depending on the environment [74]. In response to nutriment deprivation as serum starvation, it is well established that global protein synthesis is reduced. However, repression of global protein synthesis in response to stress often co-occurs with an increase in translation of some specific mRNAs encoding proteins that are essential for cell survival. As expected, in the study of Liang and colleagues, the percentage of polysome-associated mRNAs was decreased in response to serum starvation in both wild-type p53 and p53-null cells [19]. This decrease in polysome-associated mRNAs was however more important in wild-type p53 HCT-116 cells than in p53-null ones suggesting that p53 contributes in the global reduction of protein in response to serum starvation. Although the molecular mechanism by which p53 regulates global protein synthesis has not been deciphered in this work, several studies reported that p53 directly or indirectly inhibits protein synthesis in response to stress by repressing the eIFs node or the ribosome biogenesis [31]. In parallel to this global reduction in protein synthesis, serum starvation in wild-type p53 cells was associated with an increase in translation efficiency without change in transcription and a buffering effect (i.e., no alteration of translation efficiency while their transcription was drastically decreased). Interestingly, these mRNAs were enriched in mRNAs coding proteins involved in several biological processes including cell-cycle progression and regulation of cell proliferation, similarly to what observed in response to stress. These data illustrate the role of p53 in inhibiting global translation in response to stress condition that was paralleled with increased translation of pro-survival mRNAs.

In addition to affect the global protein synthesis, p53 also contributes in regulating specific mRNA translation. Interestingly, depending on the cell type, the activation of p53 by Nutlin-3a alters translation efficiency of mRNAs coding proteins involved in different biological processes. In MCF7 cells, both Nutlin-3a and doxorubicin-induced activation of p53 after 16h treatment exposure were associated with an increased translation of the mRNAs showing an uncoupled transcription/translation rate that code proteins with pro-apoptotic functions [21]. In contrast, p53 activation in immortalized human primary BJ cells was associated with a decreased translation in ribosomal genes, such as *RPL34* and *RPL23* that code ribosomal proteins [22]. Moreover, when comparing gene lists whose translation is altered without change in transcription in response to 12 h of Nutlin-3a treatment, only 0.2% was commonly altered in HCT-116, MCF7 and SJSA cell lines [20]. While no gene ontology was shown in this latter study, the mRNAs whose translation increases in treated cells are enriched in mRNAs coding proteins with zinc-finger related properties (HCT-116) or involved in histone/chromatin modeling (SJSA) (data not shown). These observations suggest that p53-associated change in translation could induce transcriptional reprogramming. One simple hypothesis can be that p53 activation quickly regulates translation of transcription-related genes to contribute in a profound but stable transcriptional reprogramming. This sequential translation/transcription regulatory mechanism would help cell in maintaining the change in transcriptional landscape induces by p53. To test this hypothesis, time-course studies based on an integrative “omic” approach will be required.

Altogether available “omic” data confirm that p53 regulates not only the global protein synthesis but also specific mRNA translation efficiency, making the proof-of-concept that p53 is a regulator of translation. However, one question is still open: is p53 regulating translation of some specific mRNAs directly or indirectly? In multiple genomic data sets generated to analyze the p53 signaling cascade, it has been shown that increase of p53-target genes at protein level starts from 6h [20]. Since a decrease in translation of mRNAs coding ribosomal proteins without change in transcription was observed from 6h post Nutlin-3a treatment [22], it remains difficult to answer this question using “omic” studies. Nevertheless, scattered pieces of evidence could be provided to try answering this question.

## 4. p53 as Direct Regulator of Translation

### 4.1. RNA-Binding and RNA-Related Properties of p53

Since the identification of p53, numerous studies have been conducted to characterize the biochemical activity of p53 protein. In parallel with accumulating evidences showing that p53 interacts with DNA, it rapidly appeared that p53 has also the capability to interact with RNA (for review see [75]). In 1991, it has been first suggested that p53 covalently binds to 5.8S rRNA through the C-terminal Ser389 residue, using murine p53-derived peptides corresponding to the last 15 C-terminal residues known to interact with DNA in a non-specific manner [76,77,78]. Later on, it has been reported using radio-labeling RNA retention assays on nitrocellulose filters that recombinant wild-type p53 purified from insect cells, binds RNA in a dose-dependent manner [79]. In contrast, recombinant p53 carrying the mutation Ala143 in the DNA-binding domain (DBD) failed to interact with RNA, suggesting that the p53 DBD is also involved in RNA-binding. Moreover, competition assays performed in presence of DNA or cytoplasmic RNA purified from the human HeLa cells revealed that p53 has a better affinity for RNA than DNA. It has also been shown using biophysical approaches and transmission electronic microscopy that addition of RNA drastically reduced the propensity of p53 DBD peptides to aggregate in vitro thus supporting the higher affinity of p53 for RNA compared to DNA [80]. Finally, yeast three-hybrid system-based screening detected p53 interactions with several different RNAs, demonstrating for the first time that p53 interacts with RNA in vivo [81]. In this study, the p53:RNA interaction was dependent upon the C-terminal domain of p53. Moreover, no specific RNA sequences or secondary RNA structures involved in p53:RNA interaction have been identified, suggesting a non-specific interaction. However, while p53 interacted with only some specific RNAs in vivo, p53 was found to bind to all of the RNAs in vitro, suggesting the specificity of p53:RNA binding occurs in vivo but not in vitro. Although technical problems have been highlighted in these different studies and exact implications of p53 DBD and OD remain to be clearly deciphered [75], altogether these studies suggested that p53 presents unspecific and specific RNA-binding properties in vitro and in vivo, respectively. 

Several studies reported that p53:RNA interaction promotes RNA base pairing of complementary sequence [79,82,83]. Recombinant p53 has indeed been shown to promote a 1600-fold increase of RNA:RNA annealing [82]. Moreover, p53: RNA interaction has been shown to contribute in the 3′ to 5′ exonuclease activity of p53 in cytoplasm that favors degradation of AU-Rich Elements (ARE)-containing mRNAs, which are usually protected by the RBP HuR [84]. Furthermore, a fraction of the cytoplasmic p53 protein has been reported to be associated with polysomes [77]. In cells, protein:RNA interaction, formation of RNA:RNA duplex but also 3′ to 5′ exonuclease activity are essential for RNA metabolism and regulation of gene expression, not only regarding mRNA splicing, mRNA stability but also mRNA translation. Thus, these biochemical studies that have been largely eclipsed at that time by the emerging DNA-related p53 properties fitting with its transcriptional activities, were only the starting point for some groups, which reported that p53 specifically interacts with some mRNAs to regulate their translation.

### 4.2. More Than a Simple RNA-Binding Protein: An IRES-TransActivating Factor?

**Figure 2:**
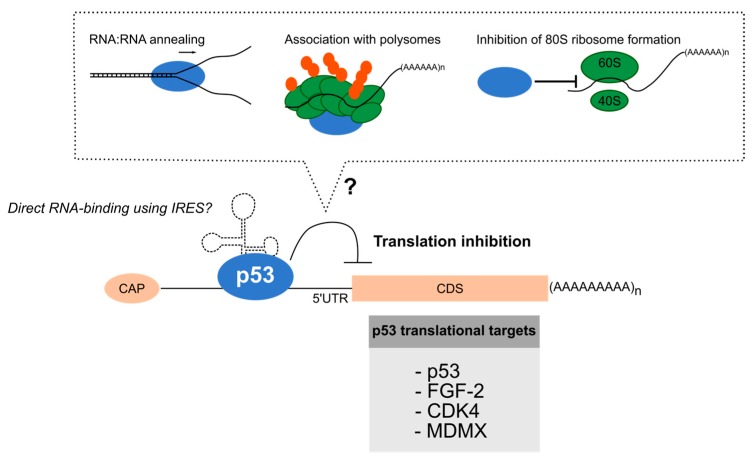
p53 interacts with the 5′UTR of some mRNAs to inhibit their translation. Direct interaction between p53 and 5′UTR of different cellular mRNAs has been identified using in vitro approaches, the p53: RNA interaction occurring in regions known to form IRES (Internal Ribosome Entry Sites). Binding of p53 on 5′UTR has been shown to inhibit translation of the related mRNAs although the molecular mechanism remains to be clarified (dotted box). It can either result from inhibition of 80S ribosome formation, presence of p53 into polysome or p53-dependent RNA:RNA annealing to favor formation of particular mRNA structures such as IRES. (AAAAA) n: poly-A tail protecting mRNA against degradation; CAP: chemical modification of 5′ mRNA end involved in binding to canonical translation factors; CDS: coding sequence of mRNA; 5′UTR: 5′-UnTranslated Region; 40S: small ribosome subunit; 60S: large ribosome subunit; blue circle: p53 protein; orange dots: amino acids of neo-synthetized proteins.

Only few studies demonstrated the direct role of p53 in translational regulation based on the RNA-binding and RNA-properties of p53 (Figure 2). The first demonstration that p53 directly regulates mRNA translation has been done on its own mRNA. In 1995, binding assays using in vitro translated and radio-labeled p53 protein and in vitro transcribed and biotinylated p53 mRNA showed that p53 protein binds to the p53 mRNA 5′UTR, which forms a stable stem-loop structure, but not with the p53 mRNA coding sequences [85]. It has to be noted that later on, the 5′UTR of p53 mRNA has been shown to form an IRES structure [86,87]. Moreover, in vitro translation assays showed that wild-type p53 protein decreased translation of p53 mRNA while introduction of mutant p53 protein or antibody-induced inhibition of p53 did not altered p53 mRNA translation [85]. Mosner and colleagues suggest that p53 binding to its own mRNA regulate its translation most likely through its RNA annealing activity. Since then, it has been reported using in vitro assays that p53 binds directly but unspecifically with four different regions of the 5′UTR of FGF-2 mRNA and inhibits FGF-2 mRNA translation [88,89]. Interestingly, p53 was able to modify the secondary structure of the 5′UTR FGF-2 mRNA that has been identified later on as an IRES [90]. It has been proposed that p53 inhibits FGF-2 translation by impairing formation of 80S ribosome. Using gel shift assays and in vitro translation, p53 has also been shown to directly bind to the 5′UTR of CDK4 mRNA through a nucleotide sequence of 36 bp to inhibit its translation [91,92]. The first demonstration that p53 can bind RNA in cell to modulate translation has been recently published [93]. Using Proximity-Ligation Assay (PLA), p53 has been shown to bind 5′UTR of MDMX mRNA. In parallel, biochemical approaches based on in vitro translation assays demonstrate that p53 binds MDMX mRNA and inhibits its translation. 

Usage of biochemical approaches allowed to clearly demonstrate the direct role of p53 in inhibiting translation. Most of these in vitro approaches were completed by the demonstration that reduction of endogenous protein level was associated with no alteration of endogenous mRNA levels. However, in these single gene-based approaches, demonstration that p53-dependent inhibition in translation occurs in cell using for example polysome profiling coupled to RT-qPCR is still lacking and remains a critical issue. Nevertheless, these data support a role for p53 in regulating translation through direct RNA-binding. In particular, a consensual molecular mechanism seems to emerge. It indeed appears that p53 specifically binds 5′UTR of mRNAs, which can form secondary structures known as IRES.

As exposed earlier in this review, it appears that some cellular mRNAs contain IRES that regulate their translation through direct binding of RBPs termed ITAFs [94]. Based on the previous data, p53 can thus be viewed as an ITAF. Interestingly, IRES-dependent translation is intimately link to stress response since it favors translation of some specific mRNAs involved in cell survival when the global protein synthesis is decreased in response to stress [95]. By repressing IRES-dependent translation in response to stress, we can make the hypothesis that p53 could avoid translation of mRNA involved in cell survival. However, the studies detailed above have been performed using in vitro assays in normal condition and the role of p53 in inhibiting their translation in stress response has never been investigated. In addition, how does p53 directly regulate translation remains to be deciphered as well as whether the RNA-related properties of p53 contribute to it. 

## 5. Indirect Regulation of Translation by p53

### 5.1. p53-Target Genes Acting as Regulator of Translation 

Forty years of research in the p53 field have identified a myriad of genes whose transcription is regulated by the transcription factor p53. Among them, a large set of genes is dedicated and/or involved in the regulation of translation that cannot be exhaustively cited here (for review see [31]). Several p53-target genes code proteins regulating the global protein synthesis. It has been shown for example that p53 regulates transcription of several genes to inhibit the eIFs node, including *eIF4E* the main component of the eIFs node or *TRIM22* [49,52,96]. Moreover, p53 inhibits the activity of the three RNA polymerases, including RNA pol I and III that synthesize rRNA and tRNA [97,98,99,100,101]. Thus, by modulating transcription, p53 can reduce global protein synthesis to maintain a low protein synthesis and thus represses tumorigenesis. It has indeed recently been demonstrating for the first time that increased rRNA synthesis is required for lung tumorigenesis driven by *KRAS*-*TP53* defects [102]. 

In addition to modulate global protein synthesis, several p53-target genes have been shown to regulate specific mRNA translation. First, p53 regulates transcription of ncRNAs involved in translational regulation, such as miRNAs and long non-coding RNAs (lncRNAs) that contribute in translation inhibition. In addition to regulate miRNA transcription, p53 has been shown to facilitate miRNA processing and their accessibility to DICER complex thus promoting their activity [103,104]. Among the p53-regulating miRNAs, the miR-34 family has been the first miRNAs identified as p53-target genes [105,106,107]. miR-34a has been shown to repress translation of several mRNAs, including for example C-Myc, Bcl-2 or Zeb [103,104]. In contrast to miRNAs, the role of lncRNAs in p53 biological activity is just beginning to emerge, although lncRNA-p21 has been shown to repress translation of JUNB and CNTNB1 [108,109]. Second, genome-wide analysis showed that p53 regulates the transcription of genes encoding RBPs, which modulate ribosome recruitment and elongation on particular mRNA [110,111,112]. For example, the p53-target gene RBM8 directly binds the HIF1α mRNA to repress its translation by decreasing the eIFs:mRNA interaction [113]. It appears today that RBPs can form different protein complexes that co-regulate distinct specific subsets of mRNAs depending on the physiological conditions thus determining cell fate [112]. From this point of view, we can hypothesise that p53 coordinates transcription of RBP sets to regulate translation of some specific mRNA subsets thus refining the p53-induced transcriptome to promote definite cell fate program. 

### 5.2. p53 and the “Specialized Ribosome” Concept 

In the translation field, it has recently emerged that, in contrast to what expected, the components of the translational machinery (ribosome and tRNA) act as direct regulator of translation [114,115,116]. In addition to regulate expression and activity of classical factors involved in translational control, p53 has also been involved in the ribosome-modulated translation. Among the p53-target genes, several ones correspond to factors that directly catalyze rRNA chemical modifications involved in translational regulation, including small nucleolar RNAs (snoRNAs) or enzymes such as *FBL* or *DKC1* [115,117,118]. For example, it has been reported that inactivation of p53 transcriptional activity using expression of large T SV40 protein or shRNA strategies increases expression of *FBL*, the 2′-O-ribose methyl-transferase of rRNA [115]. Moreover, p53 inactivation was associated with alteration of 2′-O-ribose methylation levels at some particular rRNA regions and increased in IRES-dependent translation of oncogenic-coding mRNAs, such as *IGF-1R*, *FGF-2* and *VEGF-A* [115]. Recent analysis performed using RiboMETH-seq approach, a RNA-seq-based technic to quantify 2′-O-ribose methylation levels at the 106 rRNA known sites in a single run, confirm this finding [119,120,121]. Sharma and colleagues indeed reported that levels of rRNA 2′-O-ribose methylation were altered at some given sites in p53-null HCT-116 cells compared to wild-type p53 ones [122]. Since then, the clear demonstration that ribosomes with distinct 2′-O-ribose methylation profiles directly affect both viral and cellular IRES-dependent translation has been made using purified ribosomes and in vitro translation assays [116]. In addition to the 2′-O-ribose methylation of rRNA, alteration of rRNA pseudo-uridylation catalyzed by the p53-target gene *DKC1* also modulates IRES-dependent translation of some specific mRNAs such as XIAP or Bcl-XL [123]. Recently, it has been reported that mutant p53R172H, the murine counterpart of the human p53R175H, drastically increase snoRNA expression through direct binding with the co-factor of transcription Ets2 to promote metastasis in osteosarcoma in mice models [124]. Interestingly, most of the up-regulated snoRNA correspond to SNORD and SNORA, the two classes of snoRNAs required respectively for 2′-O-ribose methylation and pseudo-uridylation of rRNA. However, the role of ribosome-modulated translation has not been investigated in this study. Altogether, these data suggest that, in addition to regulate transcription of classical translational factors, p53 also contributes in shaping the ribosome composition to modulate translation of some mRNA subsets. Interestingly, it has been shown that p53 regulates expression levels of ribosomal proteins, either through transcriptional and translational control [22,125,126,127]. However, although the impact of p53-induced change in ribosomal protein expression on ribosome composition has not been deeply investigated, we can expect to discover in the near future that p53 may also affect ribosome composition at ribosomal protein levels to regulate translation. 

## 6. Concluding Remarks

The diverse but non-exhaustive examples presented in this review give several clues contributing to demonstrate that p53 participates in translational regulation of some specific mRNAs either through direct or indirect mechanisms, including through the emerging mechanism that involves ribosome. This additional layer of regulation driven by p53 could provide novel perspectives to understand how a single protein is able to integrate the myriad of internal and external stimuli to finely regulate cell-fate by selecting the most appropriate genes in its target genes repertoire. A simple view is that in addition to induce the most adapted transcriptome to a precise biological context, p53 could in parallel shape the most efficient translational machinery and the requested *trans*-acting factors in order to refine the transcriptome at the translational levels. Thus, subtle p53-induced modifications in the expression and/or activity of translation-related factors could be sufficient to finely adapt cell-fate without drastic change in transcription thus expanding the diversity of p53-induced proteomes. Recent findings suggest that this novel view of p53-induced modulation of gene expression could also provide a mechanistic insight of the oncogenic activities of some mutant p53. The revelation coming from genome-wide approaches showing that p53 activation resulted in 75% of change in transcription but also in 25% of change in translation opens up novel opportunity to understand the molecular mechanisms given such diversity to p53 response.

## Figures and Tables

**Table 1:** Genome-wide studies supporting role of p53 in translation regulation.

Study	“Omic” Approach	Experimental Conditions	Observations	Reference
Loayza-Puch et al. (2013)	Ribo-seq	Immortalized human primary BJ/MCF7 cells + Nutlin-3a (8 mM, 6 and 19 h)	Focus on time-dependent decrease in ribosomal genes	[22]
Zaccara et al. (2014)	Polysome profiling using microarray	MCF7 cells + Nutlin-3a (10 µM, 16 h)/Doxorubicin (1.5 µM, 16 h)	About 350 genes altered at translational but not at transcriptional levels (23% of altered genes)	[21]
Andrysik et al. (2017)	Polysome profiling using RNA-seq	HCT-116/MCF7/SJSA cells + Nutlin-3a (10 µM, 12 h)	About 1000 genes altered at translational but not at transcriptional levels (36% of altered genes)	[20]
Liang et al. (2018)	Polysome profiling using RNA-seq	HCT-116 cells + serum starvation (0.1% SVF, 16 h)	About 700 genes altered at translational but not at transcriptional levels (46% of altered genes)	[19]
